# Issues Related to the Use of Visual Social Networks and Perceived Usefulness of Social Media Literacy During the Recovery Phase: Qualitative Research Among Girls With Eating Disorders

**DOI:** 10.2196/53334

**Published:** 2024-07-02

**Authors:** Elena Faccio, Margherita Reggiani, Michele Rocelli, Sabrina Cipolletta

**Affiliations:** 1 Department of Philosophy, Sociology, Education and Applied Psychology University of Padova Padua Italy; 2 Psychologist-Operator at Vivere Verde Onlus Roma Italy; 3 Department of General Psychology Padova Italy

**Keywords:** visual social networks, body image, eating disorders, risks, potentials, social networks, social network, social media, literacy, food intake, appetite disorders, appetite disorder, eating disorder, patient safety, patient-centered approach, recovery, body comparison, users, semistructured interviews, semistructured interview, girls, adolescent, adolescents, content analysis, online

## Abstract

**Background:**

The patient-centered approach is essential for quality health care and patient safety. Understanding the service user’s perspective on the factors maintaining the health problem is crucial for successful treatment, especially for patients who do not recognize their condition as clinically relevant or concerning. Despite the association between intensive use of visual social media and body dissatisfaction and eating disorders, little is known about the meanings users assign to posting or searching for edited photos and the strategies they use to protect themselves from digital risks.

**Objective:**

This study aims to examine how young women recovering from eating disorders in Northern Italy perceive the health risks and potential benefits associated with visual social networks (ie, Instagram and Snapchat). The literature has found these platforms to be detrimental to online body comparisons. It also explores the perceived usefulness, willingness, and personal interest in coconstructing social media literacy programs with girls recovering from eating disorders.

**Methods:**

A total of 30 semistructured interviews were conducted with adolescent girls aged 14-17 years at the end of their treatment for eating disorders. The following areas of research were addressed: (1) the meanings associated with the use of Instagram and Snapchat; (2) the investment in the photographic dimension and feedback; (3) the impact of visual social networks on body experiences; (4) the potential and risks perceived in their use; (5) the importance of supporting girls undergoing treatment for eating disorders in using social networks; and (6) the usefulness and willingness to co-design social network literacy programs. Content analysis was applied.

**Results:**

A total of 7 main contents emerged: active or passive role in using social networks, the impact of online interactions on body image, investment in the photographic dimension, effects on self-representation, perceived risks, self-protective strategies, and potential benefits. The findings highlight a strong awareness of the processes that trigger body comparisons in the virtual context, creating insecurity and worsening the relationship with oneself. The self-protective behaviors identified are the development of critical thinking, the avoidance of sensitive content, increased control over social networking site use, and a certain skepticism toward developing antagonistic ideologies. All these topics were considered fundamental.

**Conclusions:**

The findings provide important insights for health professionals working with youth in preparing media literacy programs. These programs aim to reduce potential risks and amplify the positive effects of online resources. They underscore the importance of addressing this issue during hospitalization to develop skills and critical thinking aimed at changing small habits that perpetuate the problem in everyday life. The inherent limitations in current service practices, which may not adequately address individual needs or impact posttreatment life, must also be considered.

## Introduction

### Background

Although intensive use of visual social media and online body comparisons have been strongly linked to body dissatisfaction and eating disorders (EDs) [1,2], the literature has only partially explored the potential impact of competence development in the mindful use of social media during hospitalization for EDs [3].

Recent research on the topic has primarily been divided into 2 main strands: one focusing on the specific ways social media is used by individuals with EDs [4,5], and the other exploring the online environment as a potential “place” for therapy [6,7]. There are no examples of collaborative projects involving mental health professionals and service users during recovery from EDs that aim to jointly examine visual social media in new ways and co-construct new awareness about its use.

The concept that the process of care is a collaborative journey involving various stakeholders—researchers, health care providers, and service users—is a principle prominently featured in official health care documents [8] but less frequently implemented in practice [9]. This principle suggests that patients are “real experts” and that their embodied knowledge can form the foundation for coproducing the care pathway. This approach presupposes that care program design occurs within the context of people’s lived experiences, including their discourses around the body, how they perceive it, and the environments in which they socialize about ideals of desirable bodies (eg, social media). It emphasizes the importance of engaging with users in their own worlds and languages, rather than being confined to the perspectives of health care professionals [9].

During hospitalization for ED treatment, girls are usually either not allowed to use mobile phones or only permitted to use them during restricted time slots. If the use of social networking sites (SNSs) is not discussed and explicitly addressed, returning home may lead patients to revert to their previous SNS usage and interpretations, potentially undermining the long-term effectiveness of the treatment. Social media literacy programs aimed at improving body image–related outcomes should be implemented not only in the context of ED prevention but also in rehabilitation [3]. According to Fitzsimmons-Craft et al [10], only 20% of therapists have asked patients with ED about the perceived impact of social media on their body image experiences. Understanding the meanings and ways in which girls use SNSs is essential for designing educational and rehabilitation pathways that help build self- and body perception based on criteria different from those that contributed to the development of the ED [11,12].

Our research represents the first step in drafting a collaborative project involving researchers, health professionals, and service users. The goal is to identify the most salient and relevant issues related to social media use from the perspective of girls undergoing treatment.

### SN Use and Body Image Concern: What Psychological Processes Come Into Play?

Social media is a significant source of sociocultural pressure regarding appearance and influences adolescents’ relationships with others as well as their perceptions of their own bodies. Active engagement in activities related to the photographic dimension on social networks (SNs) has been shown to contribute significantly to the development and perpetuation of concerns related to body image and food intake [13,14]. Such activities include publishing photographs, viewing others’ photographs, commenting or liking posts, and receiving feedback. Compared with the passive use of SN platforms, these active behaviors are more insidious in fostering dynamics of comparison and competition between bodies, thereby exposing individuals to the scrutiny of social judgment [15-17].

A clear and direct connection between posting edited photos and risk factors for EDs has been consistently confirmed by various studies [18]. Indeed, social media usage is a plausible risk factor for the development of EDs, a finding supported by research from Asia, indicating that this association is not limited to traditionally Western cultures [19].

Intensive use of SNSs has been linked to the internalization of the thinness ideal, self-objectification, and body dissatisfaction [20-22]. The literature defines the internalization of the thinness ideal as the degree to which an individual cognitively adopts socially defined standards of attractiveness and engages in behaviors aimed at approximating these ideals [20]. This process of striving for an ideal physique through the intensive use of social media can lead to body dissatisfaction and subsequently to disordered eating behaviors. Individuals may pursue an idealized body image that is often unattainable, which can persist even during recovery from EDs and increase the risk of relapse. Moreover, the habit of viewing bodies as objects on social media can socialize girls into self-objectification, as they internalize an external spectator’s perspective of their own bodies. This encourages habitual monitoring of one’s physical appearance, a phenomenon known as self-surveillance. It can lead to feelings of anxiety, reduced awareness of one’s own internal states, body shame, and a fear of internalized judgment from others. Another factor that could influence the relationship between exposure to SNSs and the development of EDs is the significance placed on feedback received through social media. Research has shown that the extent of one’s SN, as measured by the number of “friends” [23], may predict a stronger inclination toward pursuing thinness. Facebook (Meta Platforms, Inc.) use has been identified as a prospective predictor of increased symptoms related to EDs [13]. Moreover, research indicates that high “appearance exposure” specifically through Facebook, rather than overall Facebook use, is positively correlated with increased body image issues among adolescent girls [14].

### Visual SNs and the Risk of Developing EDs

Regular sharing of self-images on social media platforms such as Facebook, Instagram (Meta Platforms, Inc.), or Snapchat (Snap Inc.), along with actively manipulating these images before sharing, seems to be linked to heightened perceptions of body shape and weight, increased body dissatisfaction, and tendencies toward dietary restriction [24]. Facebook users tend to be older compared with Instagram users, and this age difference may contribute to younger Instagram users being at higher risk of developing EDs and expressing greater concerns about the impacts of sharing their images on SNs [4]. Analyzing fitness content on Instagram, particularly posts tagged with hashtags such as #fitspiration, #fitspo, and #thinspiration, reveals that the majority of images depict women with 2 prominent characteristics: thin bodies that are also visibly toned and muscular. This portrayal reinforces the notion that only a thin body can be considered fit [25]. The pairing of thinness with muscularity appears in as many as two-thirds of the photos posted by influencers [20], and these images have a more significant impact on body image concerns compared with photos posted by celebrities or models [25].

Another SNS that emphasizes the visual dimension and has experienced rapid growth is Snapchat. Currently, there is limited research dedicated to investigating the effects of this platform. One of its distinctive features is that users can apply filters to alter their appearance before sharing photos. Additionally, Snapchat is known for its privacy settings and the ephemerality of content, which is typically automatically deleted shortly after being viewed [26].

Comparative research indicates that the correlation between photo manipulation using specific editing tools (filters) and body image concerns appears to be stronger on Snapchat than on Instagram [27]. Those who frequently use Snapchat’s lenses to enhance their photos appear to experience higher levels of dissatisfaction with their appearance, leading them to seek out environments that are highly appearance-oriented and focused on enhancing their appearance. Posts on Instagram tend to be more curated, with teenagers often spending considerable time and effort choosing the “right” content before taking a photo, rather than editing it after it has been taken [28]. This investment of time and energy before capturing the photo may strongly correlate with concerns about body image compared with postphoto retouching [29,30]. Moreover, while Snapchat is often used to connect with close contacts, users may be less focused on their physical appearance when taking pictures. By contrast, the larger audience on Instagram may have a more significant influence on body image concerns.

## Methods

### Research Aims

In light of these considerations, one might inquire about how adolescent girls undergoing treatment for EDs utilize SNs. What significance do they attribute to their SN usage? Do they differentiate between manipulated and unedited images? What strategies do they use to shield themselves from the impact of the content they post or view? Are they cognizant of which modes of SN use could potentially influence clinically significant outcomes?

Our focus is on exploring the usage of purely photographic platforms such as Instagram and Snapchat, which are particularly insidious in terms of fostering comparisons between bodies on the network [27-30]. We are interested in investigating the different emotions and experiences participants have when sharing their own photos in a private and intimate network among friends, such as Snapchat [27], versus sharing them in front of a larger audience, as is the case with Instagram [30].

Another area of interest for our investigation involves understanding the meanings associated with the permanence or short duration of content created and received on social media platforms. Specifically, while content published or shared on Instagram is permanent, on Snapchat, users determine the lifespan of their content, after which it is automatically deleted [27].

Additionally, we are interested in understanding whether girls use strategies to mitigate the potential influence of exposure to certain images, whether they can differentiate between edited and unedited photos, and whether they protect themselves from uncritically accepting messages from SNs. The literature [29] has demonstrated that visual social media can also be utilized during the recovery process from an ED to promote health messages and well-being. Paradoxically, the use of SNs that involve transforming one’s own photos has also been identified as an important tool, albeit in a positive sense [26]. Therefore, it is not merely the photo itself but how it is used that determines whether it serves as an “exit” or “entry” point in managing eating behaviors.

The ability to alter one’s images has both positive and negative implications. It allows individuals to transform their appearance inexpensively and democratically, fostering a sense of personal empowerment. However, the ease of digitally altering one’s appearance also leads to an increase in the number of manipulated images that people view and are confronted with. This phenomenon may subsequently heighten dissatisfaction with one’s physical appearance and intensify the pressure to alter it.

In conclusion, we also sought participants’ opinions on the relevance of the proposed topics and what other subjects they would consider important in a collaboratively designed program focused on the responsible use of SNs, intended for potential users in the recovery process.

### Sampling and Recruitment

After sharing the objectives and content of the research with health care staff, we informed users of the local health unit for the treatment of EDs within the National Health Service in Eastern Veneto (specifically, the Casa delle Farfalle Residential Protected Therapeutic Community for Children).

The health care staff presented the research to the girls, obtained their consent, requested authorization from their parents, and provided information about the study. They also organized the schedule for appointments. All the girls who were invited agreed to participate; none declined the invitation. The participants and their families were informed of their right to withdraw from the study at any time. They signed a written informed consent form regarding their participation in the research and its potential publication.

Thirty adolescent girls, with a mean age of 15 (range 14-17) years, expressed interest in participating. The inclusion criteria were as follows: a diagnosis of an ED, being in the early stages of treatment with at least 15 days of hospitalization, having an active Instagram and Snapchat profile for at least six months before admission, and willingness to participate in interviews.

We extended the invitation to participants who were at the beginning of their treatment journey because we preferred individuals who had not yet been significantly influenced by the discussions occurring between users and health care providers during their hospitalization period.

The interviews were conducted by MRe, one of the authors of this study. After confirming their availability, she held several preliminary meetings to establish rapport and further explain the study’s objectives and implications. The interviews were conducted individually in a room on the ward where each participant met with the research assistant.

### Ethical Considerations

The research protocol was approved by the Ethical Committee of the School of Padova, University of Padova (approval number 2018/2745-10/7). Before commencing the interviews, participants and their parents were informed that participation in the research was voluntary and without any compensation, and their consent was obtained. The original informed consent that participants signed included permission to conduct secondary analysis without additional consent. The final data set is anonymized, ensuring that no identifiable private information linked to participants is included.

### Data Collection and Analysis

A semistructured interview consisting of 9 open-ended questions was designed specifically for this research (Table 1). It covered the following areas: (1) the meanings attributed to the use of Instagram and Snapchat; (2) the level of engagement with the photographic aspect and the interactivity generated around it, particularly in terms of feedback; (3) the perceived impact of Instagram and Snapchat usage on body experiences; (4) the potential benefits and risks associated with the use of SNs; (5) the importance of providing support and guidance to girls undergoing treatment for EDs in their use of SNs; and (6) the willingness to participate in and contribute to the co-design of SN literacy programs.

**Table 1:** Structure of the semistructured interview and the open questions composing it.

Areas of research addressed by the semistructured interview	Open questions that compose the interview
The meanings associated with the use of Instagram and Snapchat	How do you consider virtual reality compared with the real world? What is your main use of Instagram and Snapchat?
The investment in the photographic dimension and in the interactivity generated around it in terms of feedback	Have you ever received comments on the photos or stories you posted? How did you experience them? To what end do you publish photos or stories? Under what circumstances do you usually edit them before publishing them? In what way? What aspects do you focus on?
The perceived impact that the use of Instagram and Snapchat can have on the body experiences	When you look at your account (Instagram or Snapchat), do you feel represented by the resulting image of you? Would you like to recount a positive and a negative experience you have had with regard to Instagram, Snapchat, or both? Have you ever compared your published photos with others? What aspects did you focus the comparison on? Do you think that the information you received from the pages and the activities related to the photographic dimension had an influence on your and others’ ideas of your body?
The potential and the risk glimpsed in the use of social networking sites	Which use of the platform do you associate with an improvement in your relationship with your body and which with a worsening? How could you protect yourself with regard to use and content that could be critical in your relationship with your body? What potential and what risks do you see in the use of visual social networks?
Importance of accompanying girls undergoing treatment for eating disorders in social network use Willingness to participate in and co-design social network literacy programs	What goals should these projects on the conscious use of social media have? Would you be willing, once your treatment is over, to collaborate with the operators and share your experience as a testimonial during courses for other girls who will be hospitalized later on?

The researchers formulated the questions based on the research objectives. Initially, there were twice as many questions, but they were later streamlined to focus on the most comprehensible ones. The first interviews helped refine these questions further, addressing any areas that appeared unclear or prone to misunderstanding.

Finally, the girls were asked to provide feedback on the relevance of the suggested themes for developing programs on critical thinking regarding SN use during hospitalization. They were also encouraged to add any additional topics they considered important. The interviews took place between June and July 2021. Each interview lasted approximately 45 minutes and was audio-recorded and transcribed verbatim.

Content analysis was conducted following the guidelines outlined by Creswell [31]. The responses from participants were read and coded, creating meaning units that were categorized accordingly. This process resulted in the development of a codebook, which was then systematically applied to analyze all responses. A triangulation of analyses involving 3 researchers (MRe, EF, and SC) was conducted. This process included sharing one researcher’s initial coding to critically discuss the initial coding patterns and subsequently reach a consensus on the overall interpretation of the data. This approach allowed for interpretations to be challenged, refined, and ultimately agreed upon through collaborative discussion among the researchers.

As the analysis moved from macrocategories (general) to microcategories (specific), there was a progressive refinement and detailed coding of the responses. Initially, responses were categorized at a broader and more anonymous level, providing a general commentary. As the analysis proceeded, categories became more specific, focusing on individual experiences and emotional impacts on personal lives. For instance, the macrocategory “virtual world configuration (VW)” is subdivided into 2 categories: “VW as an extension of reality” and “VW as a world distinct from real life.” These categories represent opposite ends of a continuum that delineates how participants perceive the relationship between the virtual world and the real world. The category “virtual world as an extension of reality” is further detailed in the subcategory (self-reference) “the virtual world significantly influences self-perception.” Participants who hold this view consider the virtual world relevant, viewing it as a source of inspiration for their real-life decisions (microcategory, fourth level of analysis). Conversely, individuals who perceive the virtual world as “separate and distinct from the real world” (category—second level of analysis) specify that the virtual world “has no impact on their self-concept” (subcategory—third level of analysis) and they “regard it as transient” (microcategory, fourth level of analysis). Another example of the categorization system and codebook created can be seen in describing the use of SNSs: we classified as “active use” everything related to actively seeking contacts and interactions, engaging in discussions, sharing personal information, and editing photos and stories. By contrast, we classified as “passive use” those actions oriented toward reading and viewing content made available by others, without active personal involvement. This position entails observing rather than actively participating in the virtual scene.

The second step was quantitative: each category was assigned a code of 1 or 0 based on its presence or absence. We focused on the number of respondents who mentioned a particular category, rather than the frequency of mentions for that category. This approach allowed us to provide an overview of the girls’ attitudes toward the investigated issue. It was particularly important for us to gain a general understanding of the impact and prevalence of specific types of experiences, as well as to explore the personal meanings the girls attributed to the topic in a more qualitative manner.

After completing the analysis, all authors of the paper convened to review the results and make any required adjustments. The draft summary of the main results paper was also shared and discussed during the collaborative analysis and writing phases.

## Results

### Virtual Word’s Configuration

An overview of the results and the codebook developed during the analysis can be found in Multimedia Appendix 1.

The thematic coding of the text collected in reference to the first answer revealed 2 main categories: the first, mentioned 28 times, portrays the virtual world as an extension of everyday reality, where online activities have a tangible impact on how respondents perceive themselves. The second category, mentioned only twice, contrasts with the first by viewing the virtual world as an ephemeral and transient realm where an idealized self-image is projected. In this perspective, the impact of online activities is perceived as less significant compared with activities in “real” life, which are seen as separate and more concrete.

### SNs’ Active or Passive Ways of Use

Regarding the mode of SN use, 2 main polarities emerged: one characterized by more active engagement and the other by more passive interaction. The proactive category included initiating relationships with others (5 mentions), sharing personal narratives (11 mentions), posting personal photos or stories (3 mentions), and searching for information (23 mentions). By contrast, the passive end of the spectrum included consulting content posted by others (21 mentions). Regarding proactive use, Instagram was noted for fostering positive emotional experiences through contact and exchange with other users (3 mentions), whereas Snapchat was valued for its utility in sharing daily life and disclosing personal information (2 mentions). The most frequently mentioned activity, active information seeking through SNs, was primarily described as a source of stimulation and inspiration, driven by curiosity (13 mentions), to reinforce and update interests (2 mentions), and also as focused information seeking on body-related issues (16 mentions).

As for the eating disorder, I used it a lot to watch food videos, videos of people cooking or even people doing particular sports, gym, like, I don’t know, all those fitness posts. That was the main use.

Participant 2

Finally, the use of photo editing functions was noted. Passive use of Instagram and Snapchat refers to the reception of content generated by other users for entertainment purposes.

### Online Interactions’ Impact

The impact attributed to SNS use is expressed in terms of “social confirmation,” as they are viewed as significant for providing a channel of self-recognition (20 mentions) that includes both approval and disapproval (12 mentions). This results in emotions of personal gratification (7 mentions), support (3 mentions), and encouragement (5 mentions), but also anxiety related to the fear of judgment (5 mentions) and feelings of disconfirmation when few likes are received (10 mentions).

Back then, I thought that if more people commented on my content, that meant more people liked me. The more likes I received, the more others liked me [...].

Participant 6

The impact of online interactions varied depending on the specific use of Instagram or Snapchat (20 mentions): Instagram is perceived as having a more tangible and “real” impact compared with Snapchat (18 mentions). Feedback received on Instagram is deemed more significant, to the extent that some respondents consider it a metric for determining their personal worth (8 mentions). There was also a notable sense of distrust toward feedback gathered through SNSs (10 mentions). Consequently, receiving likes and comments was perceived as unimportant due to skepticism about the authenticity of posts. Some respondents rarely received likes and comments, feeling unpopular (6 mentions) and disregarded both on social media and in everyday life.

Regarding Snapchat, the automatic deletion of content and the narrow composition of the network seem to foster a playful, humorous atmosphere (5 mentions) and encourage creativity. This moderates the impact of feedback received (11 mentions).

### Investment in the Photographic Dimension

The use of photographs on social media platforms shifts from total involvement (24 mentions) to complete disengagement (6 mentions). Photographs serve not only for self-promotion (20 mentions), fulfilling the desire to be seen (11 mentions), and presenting the best version of oneself (7 mentions), but also for gaining a deeper understanding of oneself (9 mentions) and celebrating specific events (4 mentions). Active involvement in the photographic dimension was specifically associated with Instagram, attributed to its high degree of self-image exposure (16 mentions). SNSs are portrayed as platforms where users consistently share only the best aspects of themselves to appear perfect (7 mentions). The use of Instagram, in particular, fosters second thoughts and inhibitions regarding photo publication (10 mentions). The high degree of exposure on Instagram promotes a tendency to hyper-control the quality of photographs, induces concerns about the perfection of one’s images, and encourages individuals to scrutinize and edit their photos extensively.

When I was going to use Instagram, I never published photos [...] because many more people can see it there, even two hundred, three hundred people can see it [...]

Participant 3

The “disinvestment” contents (6 mentions) were primarily attributed to feelings of embarrassment (3 mentions) and discomfort (4 mentions). By contrast, Snapchat’s emphasis on ephemerality (3 mentions) and frivolity (2 mentions) in its content contributes to a platform dominated by carefree attitudes toward self-image. This reflects a disengagement from the photographic dimension.

### Self-Representation

The collected texts appear polarized: they describe a correlation between the body presented online and the body perceived in the real world (17 mentions), but also refute this congruence (10 mentions) in favor of emphasizing the dominance of the real. Users primarily discussed self-representation on Instagram due to Snapchat’s ephemeral nature. Sharing photographs of themselves is viewed as a means to express their ways of being and interacting with others and the world (5 mentions), as well as a method to foster deeper personal understanding (3 mentions). There was considerable emphasis on portraying a “true” image of themselves (8 mentions). In some cases, it seems feasible to accept photos from the past, particularly those depicting a thinner body, alongside acceptance of the current body image (2 mentions). However, in other cases, confronting images of oneself from a thinner period can be agonizing (3 mentions), often evoking a sense of regret. Ten responses conveyed a sense of discrepancy between the real-life body and the online representation, attributed to efforts to present oneself in a more favorable light, sometimes accentuating flaws or concealing them (2 mentions). This can lead to a perception of projecting a false self-image (8 mentions) compared with one’s subjective self-image. The existence of photos that diverge so significantly from each other also contributes to a sense of fragmentation in one’s self-image (1 mention).

### SNs’ Risks

All the collected texts addressed the risks associated with SNSs’ use, especially activities involving the photographic dimension (30 mentions). Content on Instagram, along with related interactive activities, is perceived as significantly influential in shaping ideas about the body and self-representation (21 mentions). This influence is evident in the promotion of a singular body prototype characterized by thinness, perfection, and muscularity (17 mentions). Viewing photos of others is seen as the initial step toward attaining this ideal, although it is perceived subjectively as deceptive (8 mentions). Unrealistic content shared on Instagram aims to bolster this ideal through maladaptive behaviors (11 mentions), sometimes progressing toward images that align with the “Pro-Ana” direction (5 mentions).

Many responses (24 mentions) emphasized the risk of deteriorating one’s relationship with their body due to a heightened focus on physical appearance and an increased attention to the body dimension. Participants acknowledged the role played by Instagram in exacerbating concerns about weight and body shape (11 mentions), particularly through pages where individuals provide tips related to EDs (10 mentions). The heightened concerns are perceived to induce feelings of being “wrong” (2 mentions) and body dissatisfaction (8 mentions). Comparison with images posted online is a dominant theme (19 mentions), where physical attributes, particularly thinness, are frequently cited as comparison criteria, often accompanied by feelings of envy (4 mentions) and inferiority (9 mentions).

A sort of “vicious circle” of negative influence through imitation has been described:

A famous girl puts up a picture then maybe she sees that the picture has many comments from girls who say, “You’re so thin, you’re so beautiful”, and all this, and then she continues to put up pictures of that type, and the girls obsess more and more.

Participant 4

In relation to the interactive dimension, risks are linked to social judgment and the pressure toward conformity (2 mentions). Finally, another risk identified is the underestimation and casual approach with which users engage with SNs, not fully recognizing the impact and influence that published content can have on people’s lives (5 mentions).

Regarding Snapchat, the texts indicated a more self-deprecating attitude toward sharing content and less anxiety about posting images. This makes Snapchat perceived as a platform where users feel free from concerns about their physical appearance and comparison with others.

### Self-Protective Strategies While Using SNSs

Regarding self-protective strategies, the primary defense that empowers the use of SNs without negative impacts on one’s body image is subjective critical capacity (17 mentions). This includes the ability to recognize and distinguish true content from edited ones (4 mentions) and an awareness that each person has about their own unique body (11 mentions). Useful strategies to promote this critical capacity could be implemented by the Instagram platform itself (4 mentions). One form of defense involves actively avoiding sensitive content (7 mentions), self-exposure (2 mentions), or excessive SN use (1 mention). However, some participants noted the challenge of implementing this strategy due to their strong curiosity to view certain content. The Instagram platform itself should develop protective measures regarding the generation of certain content, which could include content control (2 mentions) or content removal (2 mentions).

From the text analysis, skepticism (8 mentions) and a sense of helplessness (4 mentions) emerged regarding the lack of control over online content, as well as a feeling of inescapability (5 mentions) due to the unavoidable accessibility of certain content on Instagram’s home page, even when not intentionally sought. Additionally, other defensive strategies should involve parental controls (1 mention) and the sharing of experiences and content (3 mentions).

### SNs’ Potentials

Several potentials were identified regarding the use of SNs, especially concerning the photographic dimension. These potentials are linked to the possibility of improving one’s relationship with their body by emphasizing spontaneity and naturalness in content creation (4 mentions), which is also perceived as a protective measure against the potential for negative comments (2 mentions). The act of searching for content is perceived as supportive during difficult times (3 mentions), providing an opportunity to follow individuals who have faced similar challenges and who, having overcome them, serve as sources of support (2 mentions) and encouragement (1 mention). Platforms such as Instagram also facilitate personal expression (9 mentions) and allow individuals to take an external perspective on their experiences (3 mentions). Exploring profiles of influencers and artists who promote diverse ideas about the body offers an opportunity to discover perspectives that might not be encountered otherwise. These individuals can inspire the exploration of values that challenge prevailing trends.

Another potential lies in actively creating content that emphasizes authenticity (6 mentions) and carefreeness (5 mentions) rather than solely focusing on physical appearance or presenting oneself in an idealized manner. By promoting authentic content, individuals can contribute to limiting the proliferation of unrealistic body ideals and reducing the circulation of idealized and unrealistic representations of the body (3 mentions). However, this aspiration is tempered by skepticism regarding the actual impact of sharing “realistic” content and finding nondeceptive body ideals. Finally, some texts emphasize the potential offered by the opportunity to connect with other people (6 mentions).

With regard to the importance of guiding girls undergoing treatment for EDs to reflect on the topic, there was unanimous agreement (30 mentions), confirming all proposed topics as relevant. If the project had been implemented, the girls would have enthusiastically participated in the group, viewing it as a safe space to find the courage and express themselves (28 mentions) and being inspired by individual choices (20 mentions). To advocate for content that goes beyond the pursuit of bodily perfection, one needs to observe behaviors in others (15 mentions). Expressing oneself with humor and levity is considered the best antidote (15 mentions), but it necessitates self-confidence and a willingness to reject the sanctity often placed on one’s image (15 mentions).

The interest and willingness were confirmed for both participating in person to listen to other girls undergoing rehabilitation (30 mentions) and discussing these issues on an equal footing with peers in similar situations. Additionally, after completing the therapy course, there is interest in potentially serving as a testimonial to set a positive example of the conscious use of social media based on personal life experiences (28 mentions). This opportunity could enable several outcomes: providing support to others (15 mentions), creating a space for personal reflection (7 mentions), hearing about the challenges others have faced in overcoming EDs (8 mentions), and connecting with peers who share similar experiences (7 mentions).

## Discussion

### Principal Findings and Comparison With Prior Studies

This research investigated how young women recovering from EDs perceive both the health risks and potential benefits of visual SNs, specifically Instagram and Snapchat, which have been identified in the literature as platforms where harmful body comparisons can occur. Additionally, the study explored the participants’ willingness and interest in collaboratively developing social media literacy programs aimed at supporting individuals recovering from EDs.

The study’s findings underscore a keen awareness among participants of the mechanisms that trigger body comparisons in the online realm, which contribute to feelings of insecurity and detrimentally impact self-relationships. However, the girls do not consistently harness this awareness to their benefit. The identified self-protective behaviors include the development of critical thinking, avoidance of sensitive content, increased control over SNSs, and a degree of skepticism toward the propagation of conflicting ideologies. All these topics were deemed fundamental by the participants.

For the young people participating in the study, 3 primary modes of using Instagram and Snapchat emerged. The first mode was characterized by the relational dimension, involving affective experiences of contact and exchange, which reduced feelings of loneliness and enhanced perceptions of social support. The second mode of use focused on self-presentation and self-disclosure, particularly evident in relation to Instagram. The third mode of use involved active information seeking, specifically the search for stimuli, inspiration, curiosity, and updates related to one’s passions, interests, appearance, and eating behaviors.

The topics emerging from the data confirm findings in the literature regarding the problematic use of SNs and the development of EDs among preclinical samples. Following appearance-focused accounts on Instagram and engaging in photo-based activities, such as posting selfies or liking and commenting on photos, were associated with poorer body image outcomes [14,15,28,31,32]. Passive use characterized by role passivity, which involves the passive reception of content generated by other users for entertainment or spectatorship purposes, has been linked to a decrease in subjective well-being through social comparison [2]. Among our participants, even the passive mode of social media use—simply opening food- and body-related videos—was acknowledged by the girls as reinforcing problematic behaviors from a symptomological point of view, such as dieting and exercise obsession.

One of the most alarming findings from our research was the significant disparity between perspectives regarding the online environment either as a continuation of reality or as distinct from it (28 vs 2). Participants who viewed the virtual world as an extension of reality tended to perceive the feedback received as highly impactful on their self-perception and sense of self-worth. The significance of online feedback was closely tied to the validation it provided, through either approval or disapproval. However, some participants expressed skepticism about the authenticity of SNs, viewing the images and content as misleading. As a result, they perceived the impact of online feedback as less meaningful compared with feedback received in real life [33,34].

This finding could be pivotal for a project focused on training, discussing, and reflecting on the subject: Highlighting the distinction between individuals who trust the authenticity of the online environment and those who approach it with skepticism is an initial step toward fostering distance and increasing awareness of one’s online presence and behavior.

Self-promotion emerged as a significant motivator for engaging in photographic activities on SNs. Users aimed to portray their best selves and sought positive recognition from others, aligning with existing findings in the literature [35,36]. The study also identified other motivations such as celebrating moments or events and seeking visibility among others [2], which are enhanced by Instagram’s exposure and amplification features. Conversely, users’ disengagement was primarily driven by feelings of embarrassment and discomfort associated with self-exposure, as well as the ephemeral and light-hearted nature of content shared on Snapchat [37].

All participants reported using criteria to select photographs for publication, particularly on Instagram. Those who noted a gap between their online self-presentation and their real selves emphasized an awareness that the virtual world perpetuates a sense of falsity by showcasing only the most desirable image of oneself. Regarding risks associated with photo-related activities, Instagram was seen as a highly visual platform centered around photos, with a strong focus on aesthetic content and a prevalence of edited photos that promote unhealthy, unrealistic, and deceptive body ideals [38]. These body ideals are perceived as highly influential in promoting and striving for perfection and the thin ideal [29]. Despite often being perceived as unattainable, media portrayal of this ideal leads women to view it as normative and central to attractiveness, thereby internalizing socially constructed appearance ideals [39,40]. This internalization and subsequent comparison lead to decreased satisfaction with one’s own body, efforts to manage one’s appearance to reach this ideal, and increased concerns about body image [41]. Social comparisons upward tend to evoke envy and feelings of inferiority, in terms of not only physical appearance but also the personality and lifestyle of individuals within one’s SN. Moreover, participants emphasized the risks associated with the interactivity facilitated by SNs. This phenomenon is referred to in the literature as “body talk,” which involves interpersonal interactions focused on bodies and physical appearance [42]. Body talk reinforces the value and significance of appearance and contributes to the construction of appearance ideals [43]. Previous research has demonstrated that body talk is positively correlated with body surveillance, body shame, and perceived pressure to conform to thinness ideals [42].

In terms of the potential of SNSs, participants emphasized the significance of being able to create, choose, and share content freely. They viewed promoting active sharing of naturalistic and unedited content, characterized by authenticity and spontaneity, as a potential means to enhance their relationship with their own bodies and for personal self-expression [44]. Improving the relationship with their own bodies was also seen as potentially helpful in finding support from people who have had a similar experience. Finally, users highlighted the potential of SNs to stabilize and enhance peer relationships with previously inaccessible groups, as well as the possibility of discovering things that might be difficult to encounter in everyday offline life. Our participants also highlighted the challenge of self-discipline and avoiding potentially harmful online content. Therefore, in the context of social media literacy programs, it could be beneficial to create spaces where girls can cultivate the courage to block the use of social media in such situations, as they suggested.

Regarding defense strategies against the influence of content published on SNSs, users primarily proposed empowering subjective critical capacity. They suggested developing and enhancing social media awareness and adopting a critical approach to viewing and scrutinizing images and posts, including considerations about the realism of the images and the intentions of the posters. They also suggested enhancing the control that platforms themselves could exercise over the type and quality of images and content posted online, even advocating for automatic deletion of content that could pose risks to users’ body image. However, some users expressed skepticism and uncertainty about the feasibility and legitimacy of such controls. They believed that the best strategy was to avoid content perceived as subjectively risky for their body image and that could potentially expose their image in an undesirable way.

It should be noted, however, that recent literature suggests enhancing critical thinking about the media may not be the sole or primary mechanism of change in effective media literacy interventions. There is uncertainty about whether literacy programs aimed at reducing the risk of EDs also effectively address critical thinking about the media. Future research will need to clarify this ambiguity [45].

What emerged from our research as a whole is that girls often have an awareness of risks but struggle to use this awareness to their advantage. They can recognize edited photos but still perceive them as authentic. Only a few responses indicated that “distrust” can serve as a strategy to mitigate perceived influence. These findings suggest that enhancing critical thinking about the media may not be the sole or primary mechanism of change for effective media literacy interventions.

However, we did not explore the influence of context, particularly the role of parents and the school environment, in moderating social media use and coping with body dissatisfaction. Among the limited qualitative research available on the meanings associated with social media use, Burnette et al’s [46] study sheds light on this topic. The authors found that among adolescent girls in general (not necessarily those in treatment for EDs), parental control devices for social media and school environments may be particularly effective in helping adolescents enhance their strategies for filtering out the most harmful messages and developing a broader conception of beauty. These factors act as protective measures against the risks associated with social media use.

### Practical Implications

Given the intricate connection between SN usage and its effects on mental health [47], it is crucial to investigate this phenomenon by valuing the personal perspectives of individuals who directly experience discomfort and pain related to SN use. Health professionals should integrate discussions about SN use into their treatment of body dissatisfaction and disordered eating [2]. Much of the content emerging from the research explores new dimensions of SNS use beyond existing literature. These include identifying 3 primary modes of use, understanding the challenge of distinguishing between real and online environments and the resulting behaviors, addressing emotional detachment strategies, and considering the implications of interacting with apps that feature permanent visual content and large followings. Additionally, the study discusses platforms with temporary photo availability and intimate networks, strategies to enhance positive social media use through peer support, and methods to mitigate risks shared between users and platform providers.

Considering the ineffectiveness of prohibiting adolescents from using SNs, it could be beneficial to focus on teaching them how to navigate these environments safely instead. Rather than restricting access [48], leveraging existing SN platforms for targeted interventions is possible. This approach can utilize anonymous access to health information available on the internet, which is appealing to young people seeking such resources [49]. This strategy aims to empower youth with the knowledge and skills needed to use SNs responsibly and effectively manage their online experiences.

We believe that every problem has its solution. In other words, starting from where young people are—specifically, their desire to engage and immerse themselves in the methods and meanings of SN use—we can empower credible and interested role models (such as girls with EDs) to influence others toward more mindful use of self-protective strategies on SNs. The most impactful approach to wield this influence is to engage these individuals as influencers and collaborate with them to develop literacy programs within treatment frameworks. This research introduces fresh insights and underscores the necessity of addressing sensitive issues to design and implement more effective and tailored educational programs tailored to individual health care recipients [47].

### Limitations

Several study limitations should be noted, including the small sample size and the fact that all participants had undergone treatment at a specialized center for EDs, indicating they already had awareness of the issue. Future research could broaden the survey to include a wider range of participants, including those in the preclinical phase of EDs. This approach would enable professionals to enhance their understanding of potential histories, distress forms, and health promotion strategies, thereby guiding the management of early signs of distress. Another limitation is self-selection, as participants were invited by health care staff and chose to participate voluntarily rather than being randomly selected. This may result in a biased and unrepresentative sample of the population, as those who choose to participate may possess specific characteristics that distinguish them from the broader population. However, it is important to note that qualitative research such as this does not aim to generalize findings to the entire population. Instead, its focus is on exploring the lived experiences and meanings ascribed to those experiences by the participants themselves.

Another limitation arises from the context of ED treatment services where participants were restricted from using phones, which meant their responses relied on memory rather than current experiences. A third limitation that could be addressed in future research involves assessing the long-term retention and application of knowledge about the risks and potentials of social media use over time.

### Conclusions

While previous literature extensively covers studies on the risks of using SNs and prevention programs, our study contributes numerous ideas on addressing this topic with young people undergoing therapy for EDs by involving them as experts by experience. It allows the identification of the most significant themes that may arise, enabling health professionals to incorporate them into discussions and share the self-protection strategies identified. There is a call to leverage the potential of SNSs, such as user-generated content and interactivity, to promote beauty ideals divergent from the prevailing model, alongside content that emphasizes humor rather than appearance-centric narratives. However, the crux of the research lies not only in what to propose but also in how to propose it. Enrolling girls as coparticipants in the programs represents, in our view, the real breakthrough.

Being able to share their experiences and offer their stories as a potential source of inspiration and influence among peers allows individuals to transform from passive recipients of life, influenced by others, into conscious leaders who guide and inspire others. This involves coconstructing care pathways, creating space, and negotiating ideas with those willing to take on the new role, such as that of a patient who, after completing their treatment journey, makes themselves available to assist others at the beginning of their own journey. Co-design indeed necessitates flexibility and a strong willingness from all participants. It is not about predefined content but rather entails collaborative decision-making where meetings and individuals together determine the objectives to pursue.

## Appendix

Multimedia Appendix 1 Overview of the results and the codebook of analysis. The 4 categories are indicated from the general (macrocategories and categories) to the particular (subcategories and microcategories). The number in brackets refers to the number of respondents who evoked the relevant category upon the 30 involved. The categories were not always subdivided further; in some cases, in fact, no details were added during the interview. Interviews with young women in recovery from eating disorders (EDs) were held in North Italy in 2021.

## Abbreviations

ED: eating disorder
SN: social network
SNS: social networking site

## Data Availability

The data sets generated or analyzed during this study are not publicly available due to privacy restrictions. Indeed, they contain information that could compromise the privacy of research participants. They are available from the corresponding author upon reasonable request.
