# Supplementary material for: Issues Related to the Use of Visual Social Networks and Perceived Usefulness of Social Media Literacy During the Recovery Phase: Qualitative Research Among Girls With Eating Disorders
Source: J Med Internet Res. 2024 Jul 2;26:e53334. doi: 10.2196/53334 (PMC11252626; doi:10.2196/53334)
Supplement: Multimedia Appendix 1 [file jmir_v26i1e53334_app1.docx]

| **Macro-categories** | **Categories** | **Sub-categories** | **Micro-categories** |
| --- | --- | --- | --- |
| 1) Virtual word’s (VW) configuration  (30) | VW as a reality’s offshoot  (28) | The Virtual world has a concrete impact on the idea of self  (28) | The virtual world is relevant (13)  It inspires real life (15) |
|  | VW as apart from real life (2) | VW and exchanges as not important to define the idea of oneself (2) | The virtual world is ephemeral  (2) |
| 2) SNS’s use (30) | From an Active use | Relevance of the Relational interactions with users (5) | To keep Contact with others (3)  To share personal information (2) |
|  |  | Self-disclosure (11)  Edit photos (3)  Edit stories (3) |  |
|  | to a more Passive Use | Information seeking (23) | Inspiration & Curiosity (13)  Interest updating (2)  Project body (16) |
|  |  | Use aimed at consulting the material posted by others (21) | Spectatorship (18)  Entertainment (6) |
| 3) Online interactions’ impact (30) | Relevant for Social validation & approval (20) | (Referred to Instagram)  It confirms the Sense of personal value (8) | I received encouragement (5)  I received support (3) |
|  |  | (Referred to Instagram)  Consideration & disconfermation (12) | Anxiety (5)  Gratification (7)  Content Rethinking and deleting (6)  Disconfirm when likes are few (10)  I think I’m unpopular (6) |
|  | Not relevant, No interest in the feedback (10) | Awareness of the fact that it is normal not to please everyone (6) | Personal discretion (4)  Being appreciated is a pleasure (4) |
|  |  | Feedback is considered false (4) | Insincerity (2)  Lack of consideration (2) |
|  |  | (Referred to Snapchat) Content is deleted by default and the environment is more restricted – this makes it live with more lightness and irony what is shared (19) | Humor (5)  Carelessness (11) |
| 4) Investment on the photographic dimension (30) | Involvement &  Investiment  (24) | Self-promotion (20) | Show/be seen (11)  Show what one thinks to be the self-best version (7)  Better understanding of oneself (9) |
|  |  | Celebration or graduation events (4) |  |
|  |  | Strong impact of exposure (7)  Choice to delete photos (3) | The need to look perfect (6)  Concern (7)  Refinement of pictures (10) |
|  | Disinvestment: do not value on the photographic dimension and do not invest in it (6) | Embarrassment (3) |  |
|  |  | Discomfort to publish photos of oneself (4) |  |
| 5) Self-representation  (27) | (Referred to Instagram) Correspondence between real and virtual: one feels represented by the photo he/she posts on Instagram or snapchat  (17) | Others can get to know you better than yourself (3)  Self disclosure (5) |  |
|  |  | "True" image: the photos correspond to the way you see yourself  Promotion of real self (8) | Gratification (5) |
|  |  | Awareness of not being thinner as in the photos (2) | Regret (2) |
|  |  | The photo as a testimony of the path they are doing, lived with congruence ("that was a me of the past and I can integrate it") (3) | Congruity (3) |
|  | Discrepancy between what I am and what I show  (10) | Enhance a self-image in which one does not recognize oneself (2)  Feeling that you have falsified it (1)  Virtual world false image (8) | Not-correspondence (6) |
|  |  | Each photo is different “and you do not understand well who and how you are, in each photo you are different” (1) |  |
|  |  | Sense of precariousness of the virtual world (1) |  |
|  |  | I see myself so small in some photos (2) | Shame (1) |
| 6) Perceived Risks (30) | Promotion of a single body prototype (21) | Thinnes, muscularity and  perfection (17) | Ideal achievement Escalation: from seeing the photos to the ideal achievement (11) |
|  |  | Unrealistic contents (11) | Feeling deceived by the ideal body (8) |
|  | Body relationship worsening (24) | Concerns’ encouragement (11) | To move on to contents that goes in “Pro-Ana" direction (5)  Feeling wrong (2)  Body dissatisfaction (8) |
|  |  | Focus on the Physical appearance (6) | Excessive attention to body size (2) Editing (2) |
|  |  | Image construction linked to the sense of falsity and mystification of the ideal self (7) |  |
|  | Comparisons with others (19) | Other users look better than themselves (7) | Frustration (7) |
|  |  | Relevance of the aesthetical dimension (7) | Body surveillance (3) |
|  |  | Relevance of Thinness (14) | Envy (9)  Inferiority (4) |
|  |  | Relevance of Aspect appearance (3) | Forgetting “the real” self (3) |
|  |  | Lifestyle personality, and artistic skills, everything is lived in comparison with others and perceived with a sense of inferiority and discomfort (5) |  |
|  | Negative Interactivity (13) | Vicious circle and negative social imitation: e.g. a model publishes a photo that receives many likes which reinforces the publication of other similar contents (1) | Insecurity linked to one's own image (11)  Underestimation and lightness with respect to the impact and influence that published content can have on people’s lives (5) |
|  |  | Social judgment (6) | Conformism (2) |
|  |  | Thinnes, muscularity and  perfection (17) | Ideal achievement Escalation: from seeing the photos to the ideal achievement (11) |
| 7) Self-protective strategies  (30) | Subjective critical capacity (15) | Difficulty in discriminating between modified and unmodified contents (4) | Confusion (3) |
|  |  | Increased critical reflexivity on oneself (11) | Each body as different (4) |
|  |  | Beware of the Instagram signal that warns that the content has been changed (4)  Ensure training programmes in the use of social (8) |  |
|  | Active avoidance (8) | Avoid posting photos that are too personal (in swimmwear) (1) |  |
|  |  | Avoidance of Self-exposure (2) |  |
|  |  | Avoidance of SNS’ use (1)  Avoidance of sensitive contents (4) |  |
|  | Instagram restrictions (11) | Controls not to publish sensitive photos (2) |  |
|  |  | Content removal (2) |  |
|  | Skepticisms (8) | Difficulty deleting or refraining from not searching for certain content (4) | Helplessness (4) |
|  |  | Difficulty in eradicating the ideal of the body promoted in social media (5)  Parental Control (1)  Sharing of experiences and contents (3) | Inescapability (5) |
|  | Greater awareness of content and its influence by the publisher (2) |  |  |
|  | Parental control (1) |  |  |
| 8) Potentials (28) | SNS’s have great potential for promoting content that  reflects a certain body ideal (10) | Spontaneity (4)  Naturalness in proposing contents (4) | Protection against the possibility of receiving negative comments (2) |
|  |  | Social Support in the group (4) | Force of the group (2) |
|  |  | Source of personal expression (9) | Being oneselves (5) |
|  |  | Possibility of reflecting an external vision of oneself (3) |  |
|  |  | Search for contents perceived as a supportive with respect to the moment of difficulty faced and overcame (3)  To follow people who have faced moments similar (3) | Support (6)  Encouragement (2) |
|  | Knowledge of artists and virtual venues promoting alternative bodies (9)  Active content proposal  (10) | Authenticity (6) | Skepticism towards finding non-deceptive body ideals (2) |
|  |  | Carelessness of the ideal body (5) |  |
|  |  |  |  |
|  | Connection network (6) | Possibility to bet on something else, counterbalance the body centrality (3) |  |
|  | Connection network (6) |  |  |
| 9) Importance of accompanying girls undergoing treatment for EDs in SN’s use | Very important (30) |  |  |
| 10) What goals should these projects on the conscious use of social media have? | Literacy programmes as a place where have courage and express themselves (28) | expressing oneself with humour and lightness is the best antidote (15) | not to fall into the trap of trusting too much in camouflaged images (20) |
|  | inspired by individual choices (20). | it requires self-confidence and disavowal of the sacredness of the value entrusted in one’s image (15). |  |
| 11) would you be willing, once your treatment is over, to organise together with the operators and act as a testimonial during these courses for other girls, who will be hospitalised later on? | Yes (28) | to help other people (15)  to tell many things (7)  to hear from other people the struggles they went through to come out of the eating disorders (8)  to meet others like you (7) |  |
|  | No (2) | out of shyness (1)  out of insecurity (1) |  |
